# Supplementary material for: Proteome-wide detection of S-nitrosylation targets and motifs using bioorthogonal cleavable-linker-based enrichment and switch technique
Source: Nat Commun. 2019 May 16;10:2195. doi: 10.1038/s41467-019-10182-4 (PMC6522481; doi:10.1038/s41467-019-10182-4)
Supplement: Supplementary file 1 — Supplementary Information [file 41467_2019_10182_MOESM1_ESM.pdf]

Proteome-wide detection of S-nitrosylation targets and motifs using bioorthogonal cleavable-linker-based enrichment and switch technique

Mnatsakanyan et al.

Supplementary Figures.

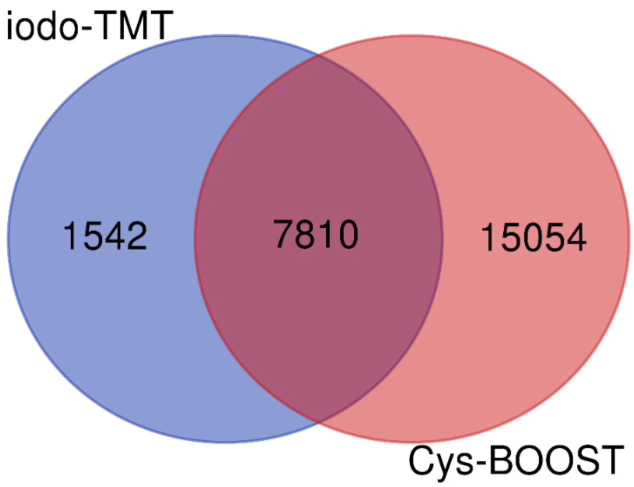

Supplementary Figure 1. Venn diagram showing the overlap of total unique Cys peptides enriched by iodoTMT and Cys-BOOST. Source data are provided as a Source Data file.

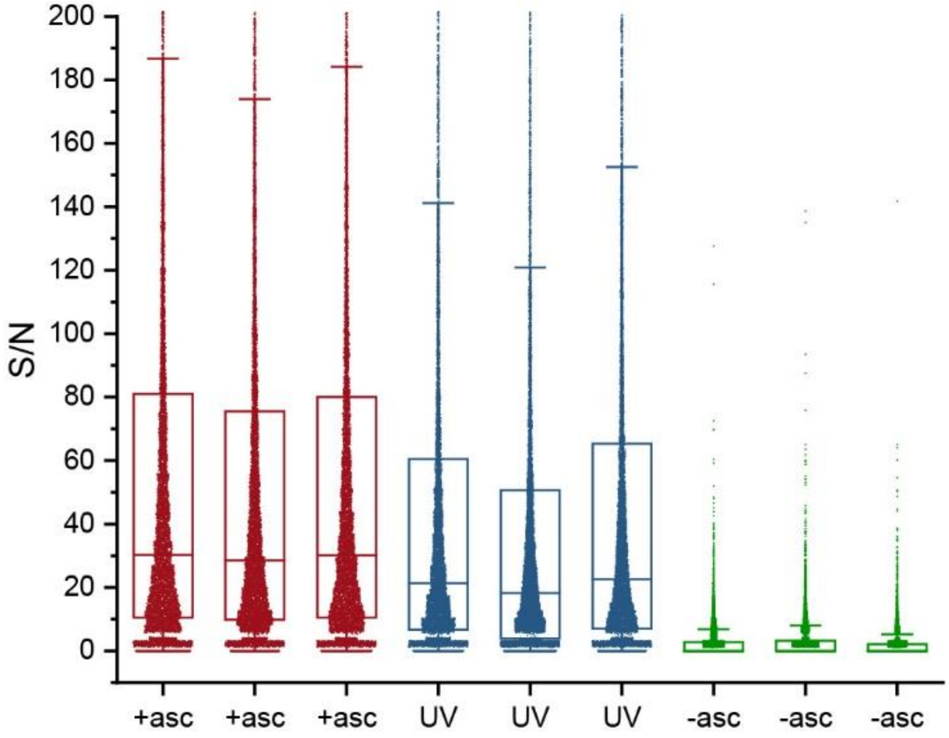

Supplementary Figure 2. Analysis of SNO reduction conditions (20 mM Na ascorbate (+asc), 10 min UV (UV) and control (– Na ascorbate (–asc)) in 200 µM GSNO-treated HeLa lysates. SNO peptides were analyzed according to the Cys-BOOST workflow. Free Cys were blocked with 100 mM IAA, excess of the reagent was removed by double acetone precipitation. SNO was switched with 3 mM IAA-alkyne (IAA-alk) in the presence (+asc) or absence (–asc) of 20 mM Na ascorbate or after 10 min exposure to UV light. After tryptic digestion, TMT labeling, enrichment and on-tip pH 10 fractionation the SNO peptides were analyzed by LC-MS3 in synchronous precursor selection (SPS) mode. The data was analyzed using Proteome discoverer (PD) v2.2. No normalization of total protein amount was applied, as the low (< 5 %) RSDs of reporter ion intensities observed for background peptides present in the Cys-BOOST flow through (1 µg measured by LC-SPS), confirm that the protein amounts in all samples were equivalent (Supplementary Data 3a). The amount of switched SNO peptides was relatively quantified in each sample based on the TMT reporter ion intensities (S/N). Boxplots of S/N of 7575 SNO peptide spectra match (PSMs) considered for quantification (average reporter S/N threshold ≥ 10, without carbamidomethylation (CAM) on Cys and oxidation on Met) show comparable S/N for +asc and UV reduction. The mean (□) S/N of the –asc samples (n=3, workflow replicates) is 2.6, the median is 0.56 (center line), with an average of 7.2 % of PSMs having S/N ≥ 10 (Supplementary Data 3b). Box limits are the 75 and 25% percentiles, i.e. the interquartile range (IQR); the whiskers correspond to the highest or lowest respective value or if the lowest or highest value is an outlier (greater than 1.5 * IQR from the bounds of the boxes) it is exactly 1.5 * IQR. Accordingly, the –asc control indicates the near completeness of the first blocking step using 100 mM IAA. The weak remaining signals in –asc samples, mostly below the limit of quantification (LOQ S/N ≥ 10), can be most likely attributed to the SNO reduction by negligible exposure to light. Source data are provided as a Source Data file.

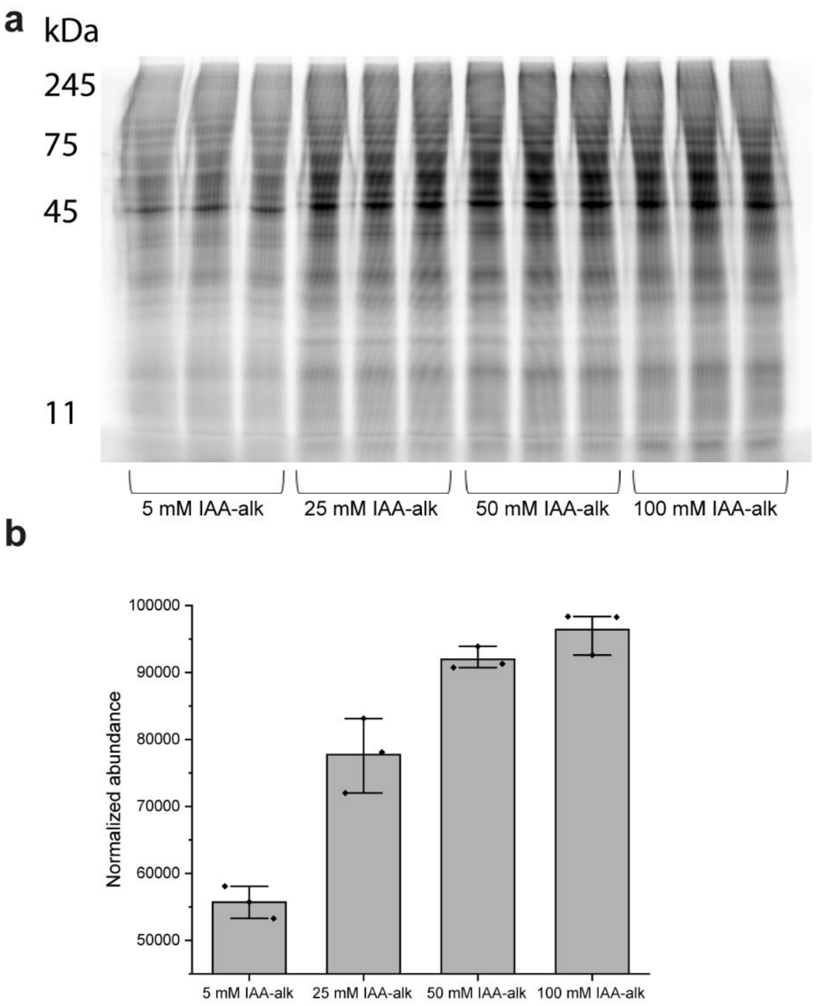

Supplementary Figure 3. Total free Cys labeling with increasing concentrations of IAA-alk. Total Cys of HeLa cell lysates (2 mg/mL) were reduced with 5 mM TCEP for 1 h at 56 °C, the pH of the samples was adjusted with 200 mM HEPES (pH 7.5), then the samples were labeled with 5 mM, 25 mM, 50 mM or 100 mM IAA-alk for 30 min at 25 °C. Excess of the reagents was removed by acetone precipitation and fluorescent 5-TAMRA-azide (Carl Roth) was introduced using CuAAC. 5 µg of each lysate were separated by SDS-PAGE, followed by fluorescence detection at 580 nm (Typhoon Trio, GE Healthcare) **a**. Afterwards, the gel was stained with Coomassie Brilliant Blue solution (gel not shown, provided in the Source Data file). Fluorescence signal intensities and protein abundance (based on Coomassie staining) were quantified using ImageJ. The fluorescence signal shows on average a 4.6 % increase in 100 mM compared to 50 mM IAA-alk labeled samples **b.** indicating the saturation of labeling at 100 mM. The flourescence signal was normalized to protein abundance (Coomassie staining) in each lane, error bars represent the minimum and maximum values of n=3 technical replicates. Source data are provided as a Source Data file.

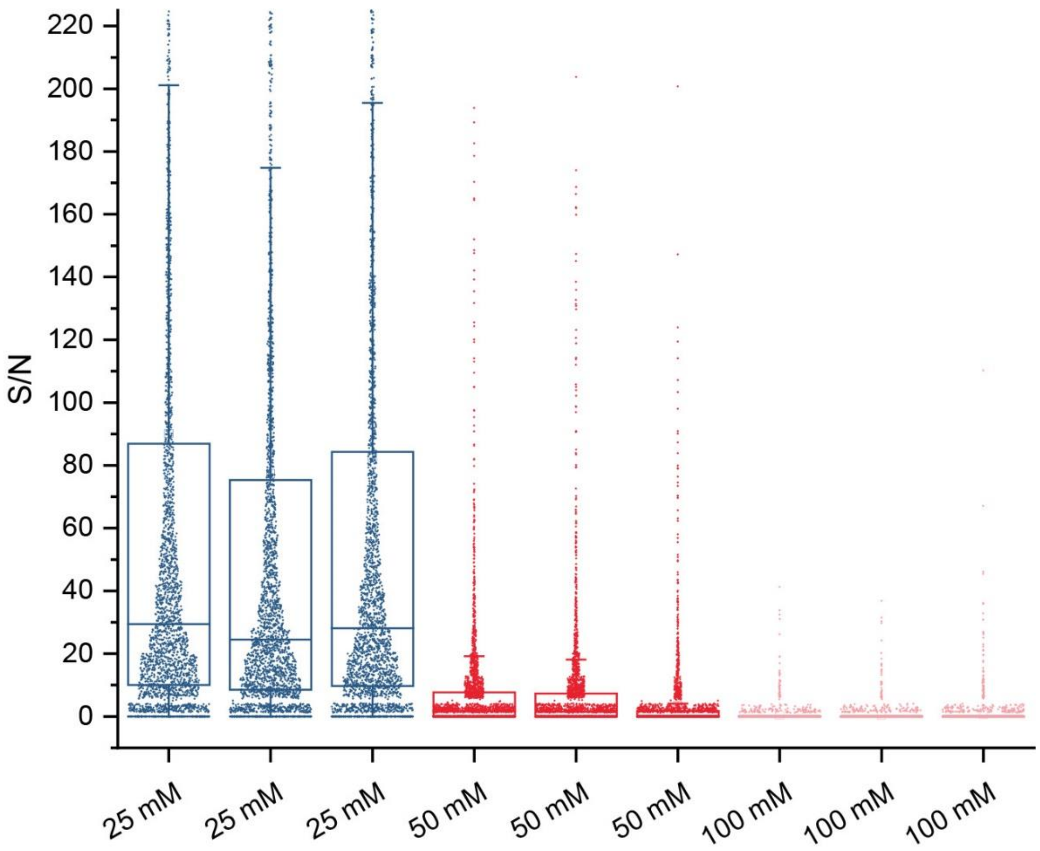

Supplementary Figure 4. Analysis of completeness of IAA blocking of free Cys in the initial step of the ST. The free Cys in SH-SY5Y cell lysates (2 mg/mL in HENS) were blocked with 25 mM, 50 mM or 100 mM IAA for 30 min at 25 °C, in triplicate. Excess of the reagents was removed by double acetone precipitation. The pellets were resolubilized and a second labeling step with 10 mM IAA-alk was applied for blocking of the remaining free Cys. After tryptic digestion and TMT labeling the IAA-alk labeled peptides were enriched using Cys-BOOST workflow. The eluate was analyzed by LC-SPS. The data was analyzed using Proteome discoverer (PD) v2.2. No normalization of total protein amount was applied, as low (< 5 %) RSDs of reporter ion intensities observed for background peptides present in the Cys-BOOST flow through (1 µg measured by LC-SPS), confirm that the protein amounts in all samples were equivalent (Supplementary Data 4a). The amount of the remaining free Cys after IAA blocking was relatively quantified in each sample based on the TMT reporter ion intensities (S/N). Boxplots of S/N of 2521 PSMs considered for quantification (average reporter S/N threshold ≥ 10, without CAM on Cys and oxidation on Met) show drastic reduction of remaining free Cys after 50 mM and 100 mM IAA blocking compared to 25 mM. The mean (□) S/N is 6.3 and 0.4, the median (center line) is 0.9 and 0, with on average 15 % and 1% of PSMs having S/N ≥ 10 in 50 mM and 100 mM IAA blocked samples respectively (Supplementary Data 4b). Box limits are the 75 and 25% percentiles, i.e. the interquartile range (IQR); whiskers correspond to the highest or lowest respective value or if the lowest or highest value is an outlier (greater than 1.5 * IQR from the bounds of the boxes) it is exactly 1.5 * IQR. Hence, confirming the completeness of the free Cys blocking with100 mM IAA. Source data are provided as a Source Data file.

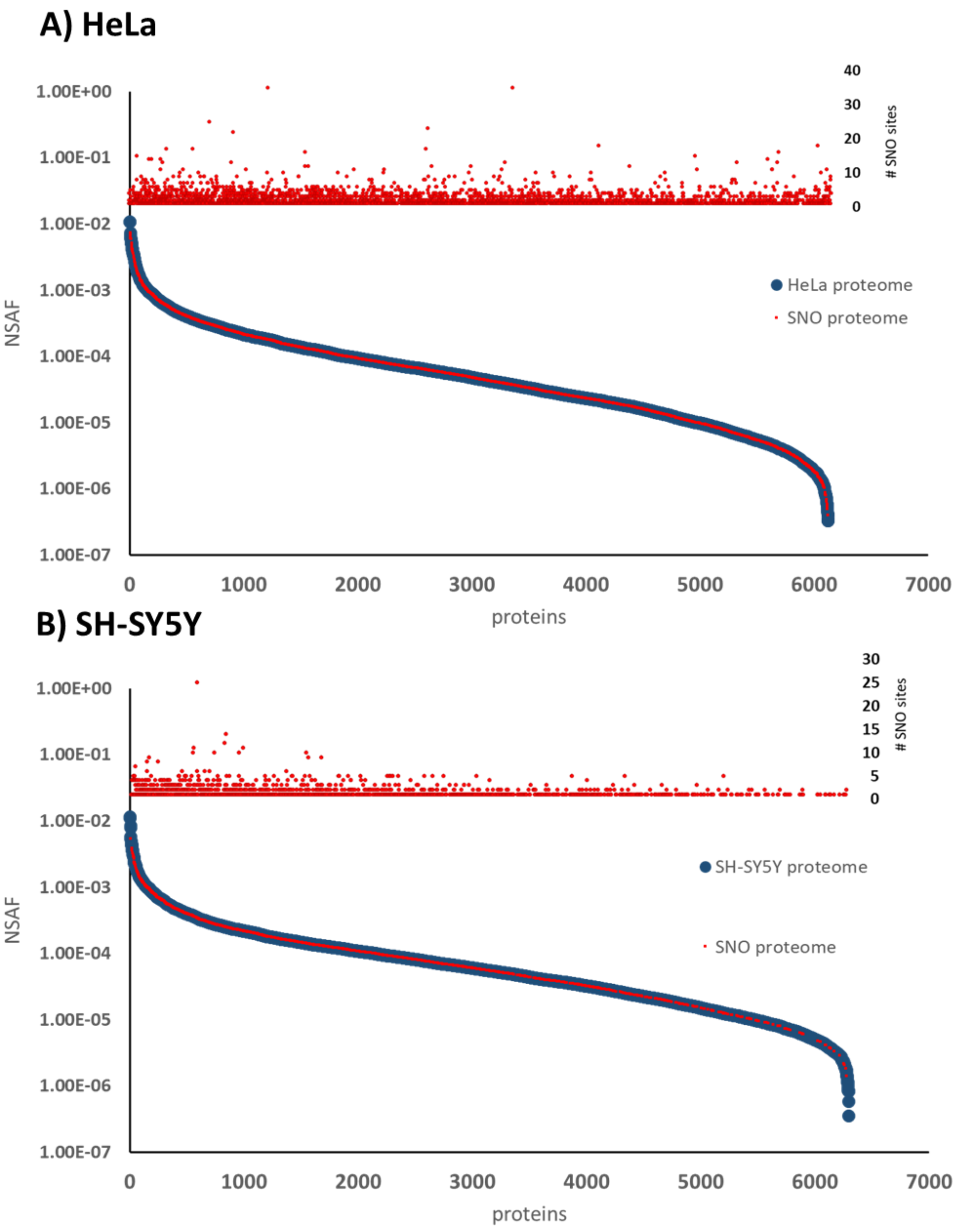

Supplementary Figure 5. Good coverage of the **a.** HeLa and **b.** SH-SY5Y SNO proteome. Proteome dynamic range represented by NSAF values (blue): Only proteins with at least 1 unique peptide (1% protein false discovery rate) were considered and ordered in descending abundance, as represented by descending NSAF values, corresponding to 6120 (HeLa) and 6294 (SH-SY5Y) proteins, respectively. SNO proteome (red): The NSAF values for proteins with quantified high confidence SNO sites are shown as red dots, corresponding to 3007 SNO proteins for HeLa and 1413 SNO proteins for SH-SY5Y. Notably, an additional 625 (HeLa) and 30 (SH-SY5Y) proteins with SNO sites are not represented in the graphs, as they were not detected with unique peptides in the global proteomes and therefore lack an NSAF value, indicating a presumably low abundance of this proteins. The quantified SNO sites clearly cover the whole dynamic range of the corresponding proteomes. Top: the number of quantified SNO sites per individual protein is plotted, representing 6247 HeLa and 2158 SH-SY5Y SNO sites. Source data are provided as a Source Data file.

Supplementary Tables.

Supplementary Table 1. SNO consensus motifs identified by motif-x v1.2 for (a) GSNO non-reactive ($R \leq 1.5$), (b) GSNO mild-reactive ($1.5 < R < 6$; p-value ≤0.05), (c) GSNO hyper-reactive ($R \geq 6$; p-value ≤0.05) SNO sites.

a. GSNO non-reactive SNO sites ($R \leq 1.5$). Mappable 480, matched 362 (75 %)

#	Motif	Motif Score	Foreground Matches	Foreground Size	Background Matches	Background Size	Fold Increase
1.	.......CL..L...	24.71	41	480	3831	315162	7.03
2.	...L...C.......	15.35	101	439	30548	311331	2.34
3.	...I...C.......	15.65	55	338	12652	280783	3.61
4.	...F...C.......	11.69	44	283	12323	268131	3.38
5.	...V...C.......	13.26	55	239	19105	255808	3.08
6.	.......CI......	9.82	29	184	9134	236703	4.08
7.	....L..C.......	7.32	37	155	20818	227569	2.61

b. GSNO mild-reactive SNO sites ($1.5 < R < 6$; p-value ≤0.05). Mappable 1,894, matched 283 (15 %)

#	Motif	Motif Score	Foreground Matches	Foreground Size	Background Matches	Background Size	Fold Increase
1.	.......C.....K.	10.53	156	1894	14849	315162	1.75
2.	.K.....C.......	6.48	127	1738	13781	300313	1.59

c. GSNO hyper-reactive SNO sites ($R \geq 6$; p-value ≤0.05). Mappable 3,446, matched 2826 (82 %).

#	Motif	Motif Score	Foreground Matches	Foreground Size	Background Matches	Background Size	Fold Increase
1.	.......C...E...	16.00	372	3446	17478	315162	1.95
2.	....E..C.......	16.00	307	3074	15468	297684	1.92
3.	...D...C.....E.	23.09	35	2767	762	282216	4.68
4.	....D..C..E....	22.12	33	2732	691	281454	4.92
5.	.......CE......	16.00	251	2699	14481	280763	1.80
6.	....D..C.......	16.00	200	2448	9916	266282	2.19
7.	.......C..E....	15.65	204	2248	12638	256366	1.84
8.	.......CD......	15.95	172	2044	10396	243728	1.97
9.	...E...C.......	12.61	182	1872	12850	233332	1.77
10.	...D...C.......	13.72	137	1690	8820	220482	2.03
11.	......DC.......	10.26	121	1553	8742	211662	1.89
12.	.......C..D....	10.51	118	1432	8709	202920	1.92
13.	.......C.....E.	9.75	125	1314	10139	194211	1.82
14.	.....E.C.......	10.28	119	1189	9752	184072	1.89
15.	.......C...D...	9.94	88	1070	6809	174320	2.11
16.	.......C.D.....	9.15	86	982	7181	167511	2.04
17.	.......CT......	6.45	86	896	8700	160330	1.77
18.	.......C.....D.	6.91	66	810	6160	151630	2.01
19.	E......C.......	6.12	69	744	7222	145470	1.87
20.	D......C.......	6.59	55	675	5324	138248	2.12

Supplementary Table 2. Table of comparison of SNO studies.

Method	Model system	Number of SNO proteins/peptides/sites	Amount of starting material/ condition [mg]	Quantification	Year	Reference
RAC	CysNO treated HEK293 cells	398 peptides	1	iTRAQ; MS2	2009	1
SNO-RAC	GSNO-treated mouse heart homogenates	951 proteins, ~2000 sites	1	Label-free; MS1	2011	2
SNO-RAC	GSNO-treated mouse skeletal muscle homogenates	488 sites	0.5	iTRAQ; MS2	2013	3
SNO-RAC	Cys-NO treated nuclear extracts of rat cortical neurons	614 proteins	0.4	N/a	2018	4
SNO ICAT	SNO-Trx1-treated SH-SY5Y cell lysate	50-76 sites	0.3	Light and heavy ICAT; MS1	2011	5
SNO ICAT	normoxic mouse heart	907 sites	N/a	Light and heavy ICAT; MS1	2017	6
iodoTMT	CysNO-treated BV-2 cells; LPS-stimulated BV-2 cells	134 sites; 101 sites	0.4	TMT; MS2	2014	7
iodoTMT	rat cardiomyocyte under hypoxia	169 proteins, 266 sites	0.3	TMT; MS2	2014	8
HPDP-biotin	CysNO-treated NPrEC cells	81 sites	1	N/a	2010	9
HPDP-biotin	LPS and IFN- γ-treated RAW264.7 cells	156 proteins	1	SILAC; MS1	2012	10
CysPAT	GSNO treated RAW 264.7 cell extracts	795 proteins, 1450 peptides	0.4	N/a	2018	11
Cys-BOOST	GSNO-treated HeLa cell extracts	3632 proteins, 8304 sites	0.3	TMT; MS2	2019	current study
Cys-BOOST	SNAP-treated SH-SY5Y cells	1443 proteins, 2158 sites	0.25	TMT; MS3	2019	current study

Supplementary References.

1. Forrester, M.T. et al. Proteomic analysis of S-nitrosylation and denitrosylation by resin-assisted capture. *Nat Biotechnol* **27**, 557-559 (2009).
2. Kohr, M.J. et al. Characterization of potential S-nitrosylation sites in the myocardium. *Am J Physiol Heart Circ Physiol* **300**, H1327-1335 (2011).
3. Su, D. et al. Quantitative site-specific reactivity profiling of S-nitrosylation in mouse skeletal muscle using cysteinyl peptide enrichment coupled with mass spectrometry. *Free Radic Biol Med* **57**, 68-78 (2013).
4. Smith, J.G. et al. Proteomic analysis of S-nitrosylated nuclear proteins in rat cortical neurons. *Sci Signal* **11** (2018).
5. Wu, C. et al. Distinction of thioredoxin transnitrosylation and denitrosylation target proteins by the ICAT quantitative approach. *J Proteomics* **74**, 2498-2509 (2011).
6. Chouchani, E.T. et al. Identification and quantification of protein S-nitrosation by nitrite in the mouse heart during ischemia. *J Biol Chem* **292**, 14486-14495 (2017).
7. Qu, Z. et al. Proteomic Quantification and Site-Mapping ofS-Nitrosylated Proteins Using Isobaric iodoTMT Reagents. *Journal of Proteome Research* **13**, 3200-3211 (2014).
8. Pan, K.T. et al. Mass spectrometry-based quantitative proteomics for dissecting multiplexed redox cysteine modifications in nitric oxide-protected cardiomyocyte under hypoxia. *Antioxid Redox Signal* **20**, 1365-1381 (2014).
9. Lam, Y.W. et al. Comprehensive identification and modified-site mapping of S-nitrosylated targets in prostate epithelial cells. *PLoS One* **5**, e9075 (2010).
10. Torta, F. & Bachi, A. Quantitative analysis of S-nitrosylated proteins. *Methods Mol Biol* **893**, 405-416 (2012).
11. Ibanez-Vea, M. et al. Characterization of Macrophage Endogenous S-Nitrosoproteome Using a Cysteine-Specific Phosphonate Adaptable Tag in Combination with TiO2 Chromatography. *J Proteome Res* **17**, 1172-1182 (2018).
