# Supplementary material for: Proteome-wide detection of S-nitrosylation targets and motifs using bioorthogonal cleavable-linker-based enrichment and switch technique
Source: Nat Commun. 2019 May 16;10:2195. doi: 10.1038/s41467-019-10182-4 (PMC6522481; doi:10.1038/s41467-019-10182-4)
Supplement: Supplementary file 3 — Description of Additional Supplementary Files [file 41467_2019_10182_MOESM3_ESM.docx]

**Description of Supplementary Files**

**File Name:** Supplementary Data 1

**Description:** Summary of Cys peptides and background peptides identified in HeLa cells using iodoTMT.

**File Name:** Supplementary Data 2

**Description:** Summary of Cys peptides and background peptides identified in HeLa cells using Cys-BOOST.

**File Name:** Supplementary Data 3

**Description:** Analysis of SNO reduction conditions.

**File Name:** Supplementary Data 4

**Description:** Analysis of completeness of IAA blocking of free Cys in the initial step of the ST.

**File Name:** Supplementary Data 5

**Description:** Summary of the Cys-BOOST data from GSNO-treated and non-treated HeLa extracts.

**File Name:** Supplementary Data 6

**Description:** Summary of the Cys-BOOST and global proteome data from SNAP-treated and non-treated (basal) SH-SY5Y cells.
