# Supplementary material for: Proteome-wide detection of S-nitrosylation targets and motifs using bioorthogonal cleavable-linker-based enrichment and switch technique
Source: Nat Commun. 2019 May 16;10:2195. doi: 10.1038/s41467-019-10182-4 (PMC6522481; doi:10.1038/s41467-019-10182-4)
Supplement: Supplementary file 10 — Reporting Summary [file 41467_2019_10182_MOESM10_ESM.pdf]

nature research

Corresponding author(s): Rene Zahedi

Last updated by author(s): Apr 15, 2019

Reporting Summary

Nature Research wishes to improve the reproducibility of the work that we publish. This form provides structure for consistency and transparency in reporting. For further information on Nature Research policies, see Authors & Referees and the Editorial Policy Checklist.

Statistics

For all statistical analyses, confirm that the following items are present in the figure legend, table legend, main text, or Methods section.

n/a	Confirmed	
☐	☒	The exact sample size (*n*) for each experimental group/condition, given as a discrete number and unit of measurement
☐	☒	A statement on whether measurements were taken from distinct samples or whether the same sample was measured repeatedly
☐	☒	The statistical test(s) used AND whether they are one- or two-sided<br>*Only common tests should be described solely by name; describe more complex techniques in the Methods section.*
☒	☐	A description of all covariates tested
☒	☐	A description of any assumptions or corrections, such as tests of normality and adjustment for multiple comparisons
☒	☐	A full description of the statistical parameters including central tendency (e.g. means) or other basic estimates (e.g. regression coefficient) AND variation (e.g. standard deviation) or associated estimates of uncertainty (e.g. confidence intervals)
☒	☐	For null hypothesis testing, the test statistic (e.g. *F*, *t*, *r*) with confidence intervals, effect sizes, degrees of freedom and *P* value noted<br>*Give P values as exact values whenever suitable.*
☒	☐	For Bayesian analysis, information on the choice of priors and Markov chain Monte Carlo settings
☒	☐	For hierarchical and complex designs, identification of the appropriate level for tests and full reporting of outcomes
☒	☐	Estimates of effect sizes (e.g. Cohen's *d*, Pearson's *r*), indicating how they were calculated

Our web collection on statistics for biologists contains articles on many of the points above.

Software and code

Policy information about availability of computer code

Data collection	Orbitrap Tribrid MS Series Instrument Control Software v2.1 SP1 and Thermo Xcalibur v4.0.27.10, Exactive MS Series Instrument Control Software v2.8 SP1 and Thermo Xcalibur v3.0.63
Data analysis	Proteome Discoverer (PD) v2.2, motif-x v1.2, STRING v10.5, Cytoscape v3.4

For manuscripts utilizing custom algorithms or software that are central to the research but not yet described in published literature, software must be made available to editors/reviewers. We strongly encourage code deposition in a community repository (e.g. GitHub). See the Nature Research guidelines for submitting code & software for further information.

Data

Policy information about availability of data

All manuscripts must include a data availability statement. This statement should provide the following information, where applicable:

- Accession codes, unique identifiers, or web links for publicly available datasets
- A list of figures that have associated raw data
- A description of any restrictions on data availability

The mass spectrometry proteomics data have been deposited to the ProteomeXchange Consortium via the PRIDE72 partner repository with the dataset identifiers PXD011131 [http://dx.doi.org/10.6019/PXD011131] and PXD012485 [http://dx.doi.org/10.6019/PXD012485]. The source data underlying Figs 2 b-f, 3 a-c, 4 a,b, 5 a-c, 6 a-c, and Supplementary Figs 1, 2, 3 a, b, 4 and 5 a, b are provided as a Source Data file.

Field-specific reporting

Please select the one below that is the best fit for your research. If you are not sure, read the appropriate sections before making your selection.

☒ Life sciences ☐ Behavioural & social sciences ☐ Ecological, evolutionary & environmental sciences

For a reference copy of the document with all sections, see nature.com/documents/nr-reporting-summary-flat.pdf

Life sciences study design

All studies must disclose on these points even when the disclosure is negative.

Sample size	No sample-size calculations were performed. Sample size was determined to be adequate based on the magnitude and consistency of measurable differences between groups.
Data exclusions	The data data originating from contaminant proteins was excluded. The rest of the data was filtered for high confidence. Only PSMs with Seach Engine Rank=1 were considered. Only unique peptides with false discovery rate (FDR) ≤ 1 % on PSM and peptide levels and for Cys peptides additionally site localization probabilities (ptmRS) of iodoTMT or cl-DDE ≥ 99 % were considered. For quantification only spectra with co-Isolation threshold ≤ 20 (for MS2 experiments, not SPS) and average reporter S/N threshold ≥ 10 were considered .
Replication	n=3 biological replicates for HeLa-GSNO and total Cys, n=4 biological replicates for SH-SY5Y.
Randomization	Randomization is not relevant for this study as the biological replicates were used merely for assessment of the reproducibility.
Blinding	Investigators were not blinded to the study groups as the experiments are based on quantitative MS data and process no subjective component.

Reporting for specific materials, systems and methods

We require information from authors about some types of materials, experimental systems and methods used in many studies. Here, indicate whether each material, system or method listed is relevant to your study. If you are not sure if a list item applies to your research, read the appropriate section before selecting a response.

Materials & experimental systems

n/a	Involved in the study	
☐	☒	Antibodies
☐	☒	Eukaryotic cell lines
☒	☐	Palaeontology
☒	☐	Animals and other organisms
☒	☐	Human research participants
☒	☐	Clinical data

Methods

n/a	Involved in the study	
☒	☐	ChIP-seq
☒	☐	Flow cytometry
☒	☐	MRI-based neuroimaging

Antibodies

Antibodies used	Immobilized Anti-TMT™ Antibody Resin (Thermo Scientific). Catalog number 90076.
Validation	Thermo Scientific Pierce Anti-TMT Antibody Resin enables selective enrichment of peptides in complex samples that have been labeled with Tandem Mass Tag™ Reagents. Specific—uses specific, high-affinity antibody for the TMT structure (Kd >100pM) [https://www.thermofisher.com/order/catalog/product/90076]. Specific Labeling, Enrichment and Quantitation of S-nitrosylated Peptides Using iodoTMT Reagents [https://www.thermofisher.com/de/en/home/life-science/protein-biology/protein-biology-learning-center/protein-biology-resource-library/protein-biology-application-notes/specific-labeling-enrichment-quantitation-s-nitrosylated-peptides-using-iodotmt-reagents.html].

Eukaryotic cell lines

Policy information about cell lines

Cell line source(s)	HeLa S3 cells were obtained from DSMZ, Germany.; SH-SY5Y cells also from DSMZ, Germany
Authentication	Morphology check by microscope. Morphology infoCells were tested negative for mycoplasma contamination.rmation was obtained from comparative observations both at high and low culture densities.
Mycoplasma contamination	Cells were tested negative for mycoplasma contamination.

Commonly misidentified lines<br>(See ICLAC register)	The used cell line is not listed in the database of commonly misidentified cell lines.
